# Memantine ameliorates cognitive deficit in AD mice via enhancement of entorhinal–CA1 projection

**DOI:** 10.1186/s12868-021-00647-y

**Published:** 2021-06-14

**Authors:** Peng Li, Jin Xu, Huanhuan Gu, Hua Peng, You Yin, Jianhua Zhuang

**Affiliations:** Department of Neurology, Shanghai Changzheng Hospital, Navy Medical University, Shanghai, 200003 China

**Keywords:** Alzheimer’s disease, Memantine treatment, Entorhinal–CA1 synapses

## Abstract

**Background:**

Memantine, a low- to moderate-affinity uncompetitive N-methyl-D-aspartate receptor antagonist, has been shown to improve cognitive functions in animal models of Alzheimer’s disease (AD). Here we treated APP/PS1 AD mice with a therapeutic dose of memantine (20 mg/kg/day) and examined its underlying mechanisms in ameliorating cognitive defects.

**Methods:**

Using behavioral, electrophysiological, optogenetic and morphology approaches to explore how memantine delay the pathogenesis of AD.

**Results:**

Memantine significantly improved the acquisition in Morris water maze (MWM) in APP/PS1 mice without affecting the speed of swimming. Furthermore, memantine enhanced EC to CA1 synaptic neurotransmission and promoted dendritic spine regeneration of EC neurons that projected to CA1.

**Conclusions:**

Our study reveals the underlying mechanism of memantine in the treatment of AD mice.

## Background

Alzheimer's disease (AD) is a progressive neurodegenerative disease, the real cause of which is still unknown. Exploring and discovering new diagnostic methods and treatment strategies are still considered to be the main challenge for AD [1]. The estimated total cost of AD in the United States in 2010 was between $159 billion and $215 billion [2]. This disease is characterized by progressive and irreversible loss of neurons in certain brain regions, and gradually affects memory, learning abilities, and language skills, causes behavioral and personality changes, interferes with the individual’s ability to perform daily activities, and ultimately leads to death [3, 4]. The development of AD is accompanied by various pathological markers and events, such as synaptic degeneration, deposition of Aβ plaques, neurofibrillary tangles (NFTs), and hyperphosphorylated tau [5, 6]. Aβ is a neurotoxic substance and is considered to be a key protein in the occurrence of AD. These changes are progress gradually, eventually leading to pathological changes in AD [7, 8]. To study the neurotoxicity mechanism results from the Aβ and finding effective drug targets always have been the hotspots and difficulties in understanding the pathogenesis of AD [9].

The entorhinal cortex (EC), an essential component of the long-term-memory system, represents the main source of input to the hippocampus and the primary target of hippocampal outputs [10]. The EC inputs to the hippocampus arise primarily from the superficial layers (II and III), while the deep layers (layers V and VI) receive hippocampal projections [11]. Disrupted functional connections between hippocampus and EC were seen in AD patients with fMRI [12]. In the early stage of AD, some excitatory pyramidal neurons in the entorhinal cortex (EC) that projection to the hippocampus are the most vulnerable to damage [13, 14]. Previous studies have shown that EC pyramidal neurons in layer II and layer III areas innervate CA1 pyramidal neurons are essential for spatial memory [15, 16]. The areas lesion of EC inputs to CA1 could resulted a spatial memory impairment [17, 18]. Although many clinic drugs are currently used to treat AD, the role of these on EC-CA1 circuits in AD remains largely unknown.

Memantine is the first pharmacologic agent approved by the U.S. Food and Drug Administration for the treatment of moderate to severe AD [19]. Memantine is believed to reduce glutamate excitotoxicity via blocking NMDA receptor-operated ion channels [20]. Furthermore, memantine acts as an antagonist at nicotinic acetylcholine receptors and at 5-HT receptors [21, 22]. Some study results also show that memantine can reduce the neurotoxicity induced by Aβ23 [23]. It also enhances the cognitive ability in different transgenic models of AD [24–26], but its neural circuit mechanism remains to be elucidated. The purpose of our research was to explore how memantine delay the pathogenesis of AD from the perspective of neural circuits.

In the present study, we used viralgenetic tools and in vitro optogenetic electrophysiology studies of the dendritic spines of EC neurons projected to CA1 and paired-pulse ratio (PPR) of optic-evoked EPSCs (oEPSCs), to demonstrate the effect of chronic administration of memantine on dendritic spine density and functional connectivity of EC-CA1 pathway in the APP/PS1mice model of AD.

## Results

### Memantine improves spatial learning and attenuates memory impairment in APP/PS1 mice

**Fig. 1:**
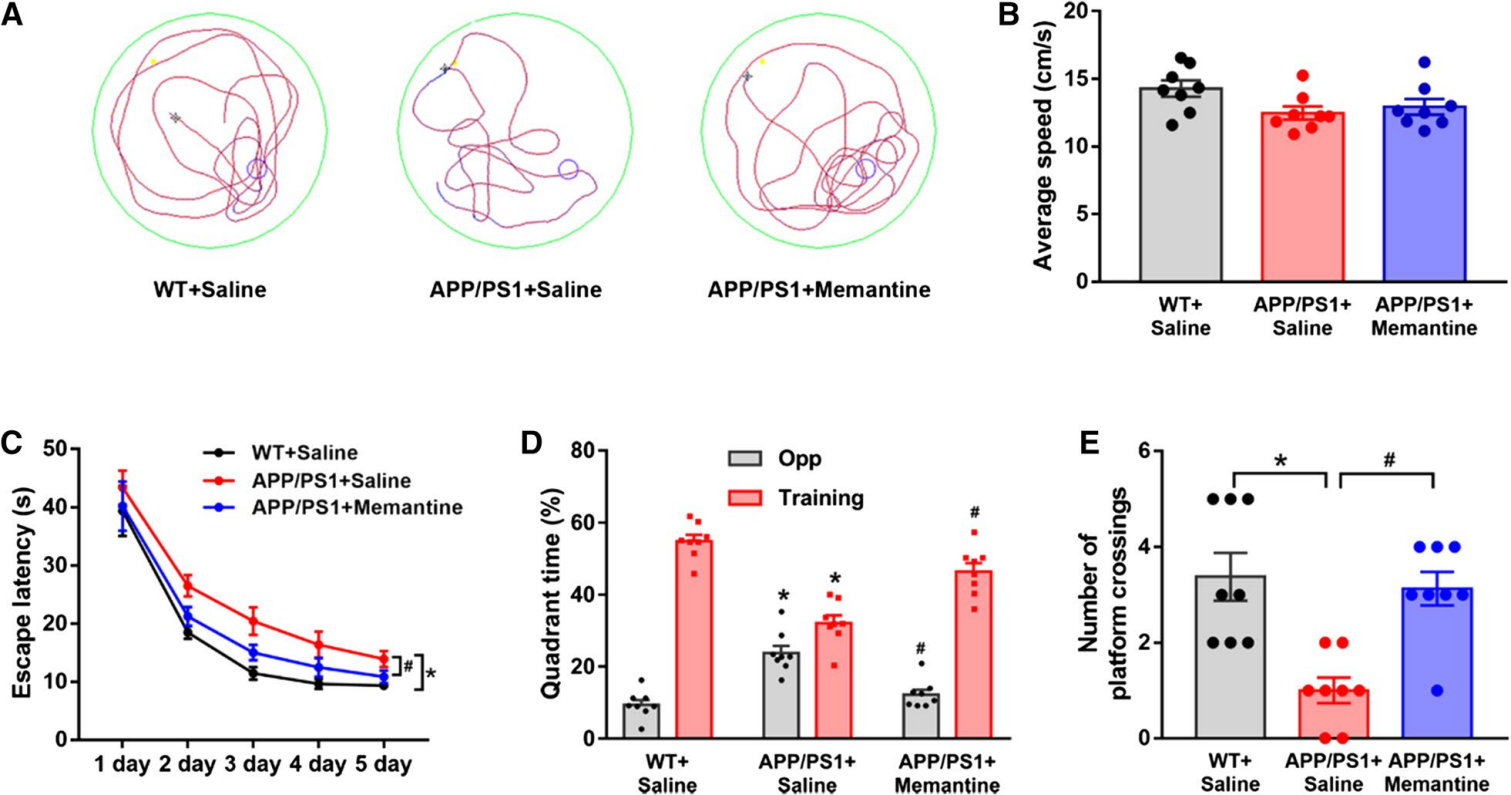
Memantine ameliorated spatial learning and memory deficits in APP/PS1 mice assessed by the Morris water maze (MWM) test. **A** The swimming trajectory of mice during the probe test. **B** Histograms showing no significant difference in the swimming speed of mice among the groups in the visible platform test. **C** The mean escape latency was given for different test days in these three groups. **D** Histograms showing that the decrease in swimming time in the target quadrant and the increase in swimming time in the opposite quadrant in the APP/PS1 + Saline group during the probe trial test was reversed by memantine treatment. **E** Number of platform site crossovers in target quadrant during probe test. n = 8 mice for per group. *p < 0.05 compared with WT mice; #p < 0.05 compared with saline treated APP/PS1 mice. One-way ANOVA, Bonferroni post-tests. Bars represent as mean ± SEM. Bar graphs show mean ± SEM

To determine whether memantine improves spatial learning and memory, Morris water maze (MWM) test was conducted in control and memantine treated APP/PS1 mice. The trace records (Fig. 1A) showed that the swimming trajectory of APP/PS1 mice in the target quadrant was smaller than those of WT mice and memantine treated APP/PS1 mice. There was no significant difference in swimming speed among these three groups (Fig. 1B). And the escape latency in different test days of training was significant for these different treatment groups (Fig. 1C). The APP/PS1 mice took more time to find the target quadrant and decreased the number of platform crossings (Fig. 1D, E). Furthermore, both parameters could be significantly improved after memantine treatment in APP/PS1 mice (Fig. 1D, E, n = 8, one-way ANOVNs, P < 0.05). Taken together, our data suggested that memantine was effective in improving the spatial memory in APP/PS1 mice.

### Memantine restores LTP in EC innervated CA1 neurons of APP/PS1 mice

**Fig. 2:**
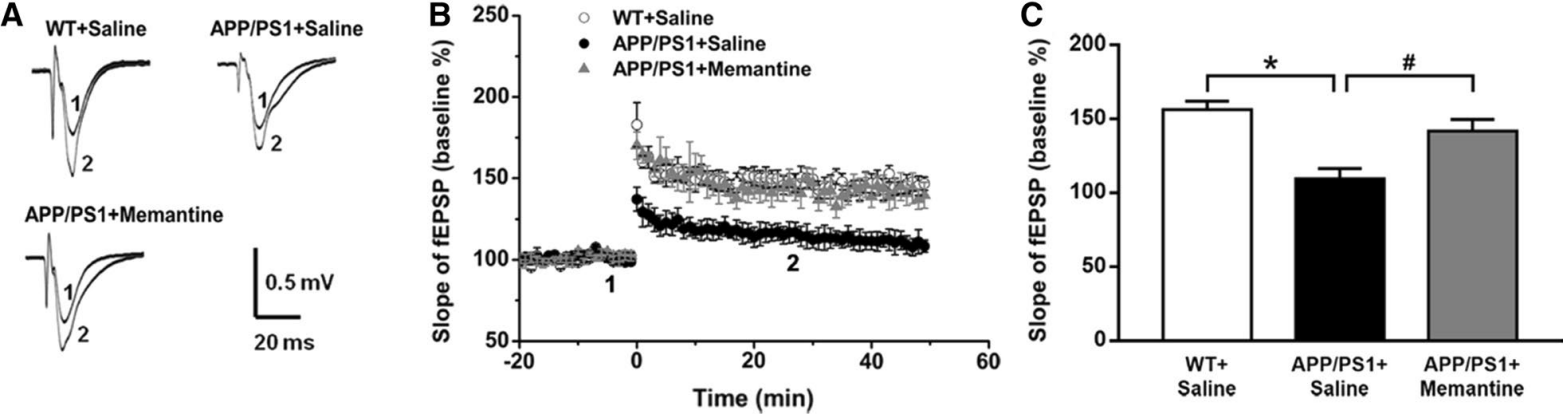
Memantine restored LTP impairment in APP/PS1 mice. **A** Typical fEPSP traces before and after TBS recorded in the different groups. Vertical bar, 0.5 mV; horizontal bar, 20 ms. **B** Time course of LTP induced in the hippocampal CA1 region of mice in the different groups (n = 6 for each group). **C** Statistics of LTP induced in the hippocampal CA1 region of mice in the different groups. ∗ p < 0.05 compared with WT mice; #p < 0.05 compared with saline treated APP/PS1 mice. one-way ANOVA, Bonferroni post-tests). Bars represent as mean ± SEM

To investigate the role of memantine on synaptic plasticity in the CA1 region of the hippocampus, evoked potentials and LTP were measured in hippocampal slices. Figure 2A showed the representative fEPSP traces before and after TBS stimulation in three groups of mice used in the present study. The time course of LTP showed that the baseline recordings were stable for 20 min, with no significant difference between WT, APP/PS1 mice and APP/P1 mice treated with Memantine. However, further recordings for 60 min following TBS revealed that the ratio of LTP was significantly reduced in APP/S1 mice compared to WT mice (Fig. 2B). The depression of LTP 30 min after TBS from APP/PS1 mice treated with Memantine confirmed that memantine significantly enhances the magnitude of the LTP (Fig. 2C). The average fEPSP slopes of LTP for the different groups was as follows: WT + Saline, APP/PS1 + Saline, APP/PS1 + Memantine (Fig. 2C). These data confirm that memantine can rescue impaired LTP in mouse of APP/PS1.

### Memantine prevents dendritic spines degeneration of EC neurons projected to CA1 in APP/PS1 mice

**Fig. 3:**
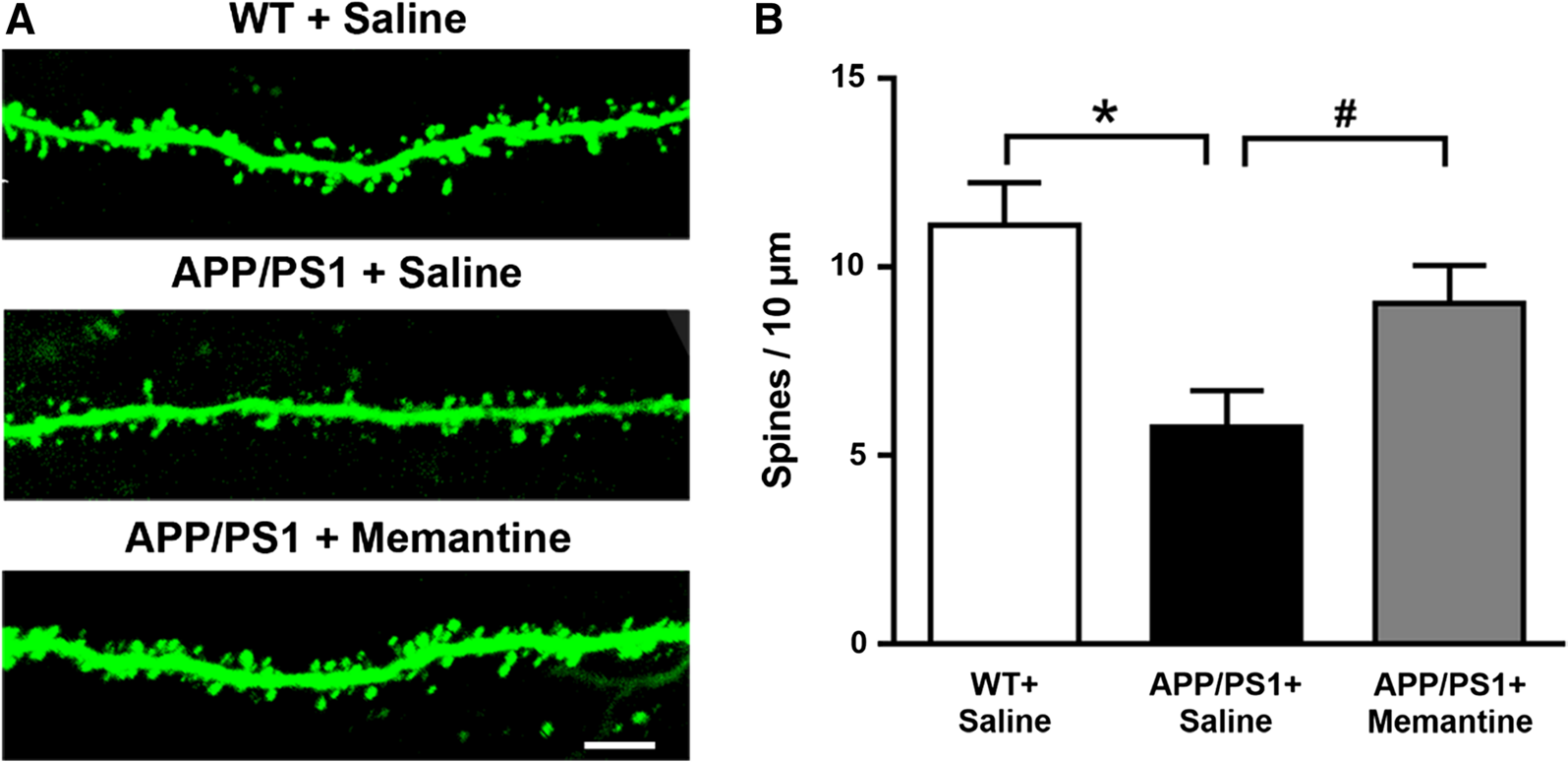
Memantine improved the dendritic spines of EC neurons projecting to CA1 in APP/PS1 mice. **A** Representative spine morphology of EC neurons in WT + Saline, APP/PS1 + Saline, APP/PS1 + Memantine mice. Scale bar: 5 μm. **B** Quantification of the densities for total spines. n = 15–20 neurons (from 3–5 mice) for respective groups, respectively. ∗ p < 0.05 compared with WT mice; #p < 0.05 compared with saline treated APP/PS1 mice. Bonferroni’s post hoc test following one-way ANOVA. Data are shown as mean ± SEM

It was reported that the deficit of EC dendritic spine density was partially rescued by chronic memantine treatment in fragile X syndrome (FXS) and syndromic autism models [27]. Therefore, we hypothesized that memantine could increase dendritic spine density of EC neurons within EC-CA1 circuit in APP/PS1 mice. To test our hypothesis, we injected CAV-Cre virus, which allowed Cre to be delivered at a specific region by canine adenovirus type 2 (CAV hereafter) that efficiently transduces axon terminals [28, 29], and Cre-dependent AAV-DIO-EGFP virus, into CA1 and EC, respectively. This method specifically labeled the EC neurons projected to CA1. 10–15 days after the injection, dendritic spines of EC neurons projected to CA1 were also labeled by intracellularly injected fluorescent dye Lucifer yellow. Statistical analysis revealed a significant decrease in dendritic spine density of EC neurons projected to CA1 in APP/PS1 mice, and the loss of dendritic spines could be restored by memantine treatment (Fig. 3).

### Memantine improves the functional degeneration of the EC–CA1 pathway in APP/PS1 mice

**Fig. 4:**
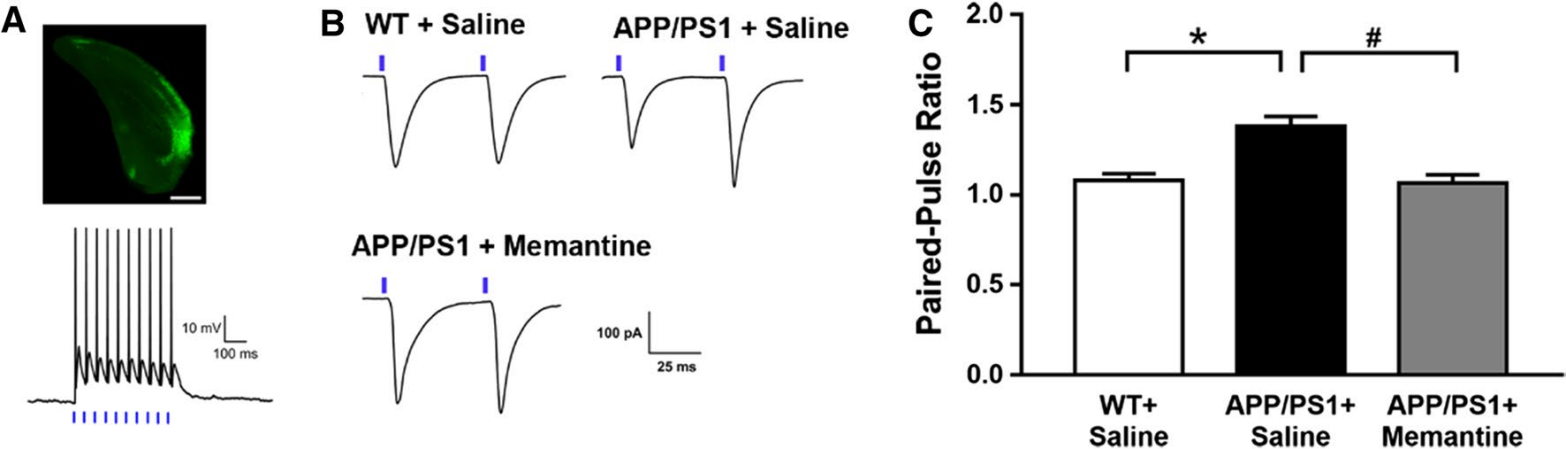
Memantine attenuated the decreased PPR of the EC–CA1 pathway in APP/PS1 mice. **A** Representative injection site of AAV-ChR2-EGFP in EC area and a representative trace of blue light-evoked action potentials during whole-cell recording. Scale bar: 1 mm. Vertical bar, 10 mV; horizontal bar, 100 ms. **B** Representative traces of oEPSCs upon paired light stimulations with 50 ms intervals. **C** PPR with 50 ms intervals of WT + Saline, APP/PS1 + Saline, APP/PS1 + Memantine mice. n = 15 neurons (from 3–4 mice) for respective groups, respectively. ∗ p < 0.05 compared with WT mice; #p < 0.05 compared with saline treated APP/PS1 mice. Bonferroni’s post hoc test following one-way ANOVA. Data are shown as mean ± SEM

Then, to complement our morphological findings which show defects in synaptic structure in vivo, we examined the functional properties of memantine on EC–CA1 pathway. AAV-CaMKIIα-ChR2–EGFP virus was stereotaxically delivered into EC. The expression of ChR2–EGFP was observed after the injection of the virus into the EC (Fig. 4A). Animals were kept for minimum of 6 weeks after virus injection in order to have sufficient opsin accumulation in the axons in the CA1. The brain sections were used to perform in vitro optogenetic and electrophysiological recordings. Whole-cell recording was made in CA1 neurons and optical stimulation at the fibers from the EC to the CA1 produced excitatory postsynaptic currents (EPSCs) in CA1 neurons. To investigate the effect of memantine on EC–CA1 pathway, we measured the paired-pulse ratio (PPR) of light-evoked EPSCs in three groups. PPR, measured by the ratio of EPSC amplitude in response to two successive stimulation pulses, reflects the facilitation of presynaptic release probability, a higher PPR correlates with lower release probability [30]. We next measured PPR of optic-evoked EPSCs (oEPSCs) to determine whether EC-CA1 synaptic plasticity could be restored by memantine in APP/PS1 mice. Figure 4B was typical traces recorded from CA1 neurons after the optical stimulation at the EC-to-CA1 fibers in response to 2 ms light pulse in different groups. Notably, from these raw traces, it appeared that the APP/PS1 mice showed a larger PPR of oEPSC and the memantine treatment could reduce the value. The average PPR for the different groups was as follows: WT + Saline, APP/PS1 + Saline, APP/PS1 + Memantine (Fig. 4C). These results suggest that the efficacy of neurotransmission from the EC to CA1 is decreased in APP/PS1 mice, and this, at least in part, account for the spatial learning and memory impairment. Memantine may improve the cognitive impairment of APP/PS1 mice through enhancing this EC–CA1 pathway.

## Discussion

It is extremely difficult to develop treatment methods for specific causes of individual cases. Because AD is a highly heterogeneous disease caused by a variety of genetic and environmental factors. In our study, we showed that memantine was effective for improving cognitive capabilities and restoring LTP impairment in APP/PS1 mice. Furthermore, we used CAV-Cre virus labeling techniques to verify the deficits in dendritic spines of EC neurons projected to CA1. Functional connection between EC and CA1 was measured in *vitro* by optogenetic stimulation combined with eletrophysiological recordings. To the best of our knowledge, this is the first study to report a specific neural circuit of EC-CA1 pathway under the influence of memantine treatment in AD-related mouse model.

Previous studies suggested that excitatory pyramidal neurons in the entorhinal cortex, which primarily target the hippocampus, are the most vulnerable brain cells in the early stages of AD [31–33], and the morphology of EC dendritic spines were disrupted in AD mice which was identified by Golgi-Cox staining [34]. However, it was not clear how the morphological changes in dendritic spine correlate with specific neural circuit in AD model mice. One recent study showed that the dendritic spines of EC neurons, projecting to CA1, were dramatically decreased in APP/PS1 mice, and memantine treatment could restore the deficit. It is speculated that memantine could improve spatial memory impairment through EC-CA1 synapses. In future study, we will focus on the correlation between spatial memory and EC-CA1 pathway in AD model mice, which would be interpreted as the underlying mechanism of memantine in clinical application.

Previous studies have shown that long-term treatment of AD with memantine can ameliorated the learning and memory deficits as well as neuropathological changes in transgenic AD mouse models [23, 24, 35–41]. Some reports are very relevant to our research. Treatment of AD with memantine reduce the amount of insoluble Aβ partly through NMDA receptors and then restore the glutamate homeostasis in animal models of AD [42–44]. Chronic oral memantine treatment can reduces hippocampal CA1 neuron loss and restore learning and memory performance [37]. Our research results also confirm that memantine improves cognitive behaviors in AD mice, which is consistent with previous reports. In ours study, we have only conducted a MWZ test, and other research used a variety of cognitive assessment methods such as open filed, new object recognition [37], step-down passive avoidance test, shuttle-box test [36]. These cognitive behavioral tests reflect different aspects of cognition. The MWM test was mainly used to evaluate spatially related memory, and did not sensitive to testing of working memory. Other studies have shown that amyloid deposition accumulated in the brain of AD mice at 8 months of age, whereas synaptic transmission from EC to CA1 were abnormal at 6 months of age [13]. These findings indicated that neural degeneration is irrelevant with the presence of amyloid plagues. In accordance with this, our findings show that the functional destruction of EC-CA1 pathway could be restored by memantine in at 6 months of age in APP/PS1 mice. However, future studies are required to explore the role of this specific neurocircuits in the improvement of cognitive behaviors by memantine. And the molecular mechanism underlying degeneration of EC-CA1 synapses by memantine in early-stage of AD reminds to be determined.

## Conclusion

In summary, we observed distinct beneficial effects of memantine in the treatment of AD. While this study identified novel mechanisms of memantine (i.e. impact on EC-CA1 circuits) which are independent of the well-versed NMDA pathways, further clinical research is guaranteed to establish the impact on disease modification in humans.

## Methods

### Animals and treatment

APP/PS1 mice purchased from the Shanghai Model Organisms Center, Inc. of China. APP/PS1 mice were in B129S background and were genotyped as reported. All experimental procedures involving mice were carried out in accordance with international guidelines on the ethical use of animals at the Changzheng hospital. The mice were housed in groups of three to five per cage under a 12 h light/dark cycle (7:00 AM to 7:00 PM) in a temperature (21 ± 1 °C) and humidity (50 ± 5%) controlled environment. APP/PS1 mice used in this study were identified as homozygous. Memantine or saline was administered by intragastric administration (20 mg/kg body weight) at 9:00 am once daily for 2 months. Mice in every group were age-matched and gender-matched. In the present study, male mice weighing between 28 and 35 g were used to morris water maze behavior and all other experiments. Animals were euthanized by decapitation after inhaling 5% isoflurane at the end of the behavioral tests. All the experiments and analyses were performed regardless of genotype or treatment.

### Morris water maze test

Morris water maze (MWM) test was performed according to the protocol described in our previous study [35]. 8 male mice were using in each of the three groups. During training period, platform was located in the same position (one of four quadrants of the pool), and the mouse was placed into the pool facing the wall at one of the four start positions, and its movement was tracked by digital tracking system. The animal was immediately removed from the water when it located the platform. If the mouse did not locate the platform after 60 s of swimming, it was gently guided to the platform or placed on the platform for an additional 15 s before being removed from the pool. The animal was tested in four trials per day with an inter-trial interval of approximately 30 min. The mice were trained for 5 days. The probe trial was performed on the sixth day following the last training session. During the probe trial, the platform was removed from the pool and the mouse was placed in the pool facing the wall from the diagonally opposite side of the platform. The mouse was allowed to swim freely for 2 min with digital movement-tracking recorded by computer software (EthoVision software) before it was withdrawn from the pool.

### Stereotaxic surgery

Mice were subjected to the operative procedure using aseptic technique. For monosynaptic retrograde tracing of EC pre-synaptic neurons, AAV-DIO-EGFP or AAV-CaMKII-ChR2-EGFP was injected into EC (2.9 mm posterior and 3.2 mm left or right to the bregma, and 4.3 mm deep into the skull surface). At the same time, CAV-Cre was injected into CA1 (1.45 mm posterior and 1.6 mm left or right to the bregma, and 1.6 mm deep into the skull surface). After injection, the hand-held needle was kept in place for an additional 5 min to avoid backflow. Following injection, the mice were collected on a 37 °C warm plate for recovery. Mice were allowed to recover for 10 day or 6 weeks before starting the experiments [45].

### Intracellular injection of the fluorescent dye lucifer yellow

The Intracellular injection was conducted as previously described [46]. Three groups of wild type, saline treatment and memantine treatment were use 3, 3 and 5 male mice, respectively. After mice recovered, the brains were fixed by 4% paraformaldehyde and 0.1–0.2% glutaraldehyde phosphate buffer (pH 7. 4) and brain slices were sectioned on a vibratome at thickness of 200 μm. The neurons in EC were loaded iontophoretically with a 4% Lucifer Yellow solution (Sigma Aldrich, St Louis,MO), using sharp micropipettes with a negative current of 3 nA (EPC10, HEKA, Germany). Three slices per mouse were injected with Lucifer yellow and 5–6 cells were injected per slice. The brain slices were analysed by two-photon imaging (Olympus PVMPE-RS) with 60 × lens. The Z interval was 5 μm. Then, the diameter of spine head and neck and the length of spines and dendritic shafts were measured with NeuronStudio. The number of spines per micrometer along the dendritic longitudinal axis was defined as the spine density.

### In vitro electrophysiology

The electrophysiology was conducted as previously described [47]. Brains were dissected quickly and placed in the artificial cerebrospinal fluid (ACSF) containing: 125 mM NaCl, 2.5 mM KCl, 2 mM CaCl_2_, 1 mM MgCl_2_, 25 mM NaHCO_3_, 1.25 mM NaH_2_PO_4_, 10 mM Glucose and saturated with 95% O_2_ and 5% CO_2_ at ~ 0 °C. Coronal brain slices (300 μm thick) were prepared with a vibratome and transferred to a chamber at 31 °C. Slices were incubated for at least 1 h before patch-clamp recording. Neurons were targeted for whole-cell patch-clamp recording with glass electrodes having a resistance of 5–8 MΩ when filled with the patch pipette solution. The electrode internal solution was composed of 115 mM CsMeSO_3_, 10 mM HEPES, 2.5 mM MgCl_2_, 20 mM CsCl_2_, 0.6 mM EGTA, 10 mM Na phosphocreatine, 0.4 mM Na-GTP and 4 mM Mg-ATP. 3 male mice were using in each of the three groups for LTP recording. fEPSPs were recorded in the CA1 while a concentrated stimulating electrode were placed in Schaffer-collaterals. LTP was induced by 3X theta burst stimulation protocol (TBS; 4 pulses at 100 Hz repeated with 200 ms inter-burst intervals). To determine whether the magnitude of LTP differed significantly among groups, the average fEPSP slopes of 30 min after LTP induction from each group were compared. Data were acquired and analyzed by using pClamp10.7 and Clampfit 10.7 (Axon Axopatch 700B, Molecular Devices, US). The experimenter was blinded to the genotypes and treatments.

For the optic-evoked paired pulse experiment, three groups of wild type, saline treatment and memantine treatment were use 3, 3 and 4 male mice, respectively. 300 μm coronal slices containing the CA1 were prepared from mice expressing AAV-CaMKIIα-ChR2-EGFP in terminals of EC pyramidal neurons. All cells were held at − 70 mV under voltage-clamp mode. Photostimulation (2 ms pulses of 1–2 mW, 473 nm light delivery via LED through a 40 × microscope objective, pulsed 2 ms of light separated by 50 ms) was used to stimulate terminals of EC pyramidal neurons expressing AAV-CaMKIIα-ChR2-EGFP. The light intensity of the LED was not changed during the experiments.

### Statistical analysis

Numerical data were expressed as mean ± SEM. Off-line data analysis was performed using software Clampfit (Axon Instruments, USA) and GraphPad Prism 6 (GraphPad Software, USA). Statistical significance was determined by ANOVA followed by Bonferroni post-tests for multiple comparisons among more than two groups. In the electrophysiology studies, n refers to the number of cells. Every group of cells in each experiment was from at least 4 animals. For all results, p < 0.05 was accepted as being statistically significant.

## Data Availability

The datasets used and/or analysed during the current study are available from the corresponding author on request.
